# Effect of Chemical Debridement and Irrigant Activation on Endodontic Treatment Outcomes: An Updated Overview

**DOI:** 10.7759/cureus.21525

**Published:** 2022-01-23

**Authors:** Nesreen Tashkandi, Faisal Alghamdi

**Affiliations:** 1 Endodontics, Faculty of Dentistry, King Abdulaziz University, Jeddah, SAU; 2 Oral Biology, Faculty of Dentistry, King Abdulaziz University, Jeddah, SAU

**Keywords:** review, outcome, intracanal irrigants, endodontics, debridement

## Abstract

Chemical debridement is considered one of the most important steps during root canal treatment to target unreached areas and provide thorough disinfection of the canals. The efficiency of this step efficiency can be increased using different agitation and irrigation techniques/devices. This comprehensive review aimed to summarize the effect of various irrigant activation and agitation techniques/devices on endodontic treatment outcomes. Using mechanical active irrigation, which enables the activation or agitation of the irrigating solution, is beneficial in root canal treatment by increasing the efficiency of delivering the irrigant up to working length and ensuring isthmus cleanliness. However, considerable variation was noticed between the protocols used in each technique with a lack of well-designed randomized clinical trials to focus on the long-term outcome. Moreover, a low level of evidence was noticed regarding the effectiveness of certain activation techniques over others. Therefore, each study needs to be carefully weighed before using its results and embracing its conclusion. Future studies need to focus more on the antimicrobial effect of each technique and its effect on the healing of apical periodontitis. Also, recent advances, such as multisonic and laser activation, are promising tools that need more clinical investigations to show their efficiency.

## Introduction and background

**Figure 1:**
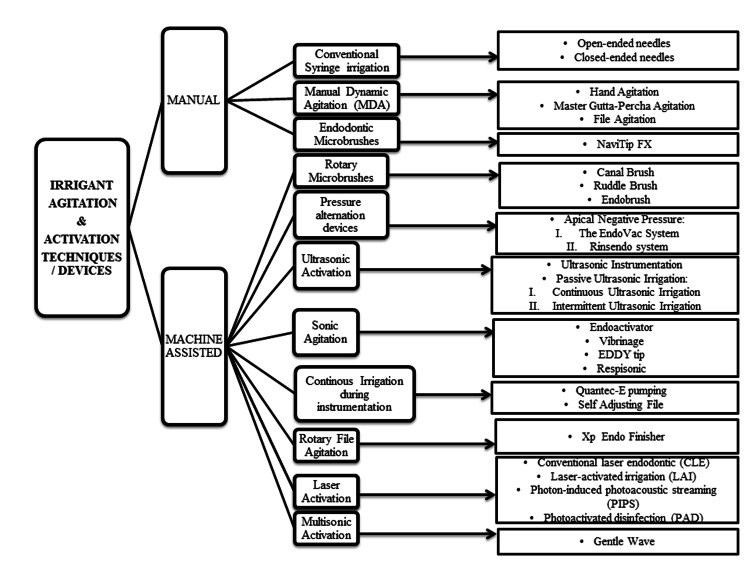
Flow chart of different irrigant activation and agitation techniques/devices

Chemical debridement is achieved by effectively cleaning and disinfecting the root canal system using irrigants and appropriate techniques. It has an essential role in the success of root canal treatment [1]. The complex anatomy of the root canal includes fins, isthmuses, lateral canals, accessory canals, and anastomosis, leading to considerable missed areas during mechanical instrumentation [2]. Moreover, the bacterial biofilm, viruses, yeasts, archaea, and smear layer formed during instrumentation make the chemical debridement procedure more challenging and form a major obstacle to completely cleaning the root canal system [3]. Irrigants used in chemical debridement should have the ability to penetrate dentinal tubules and offer a strong, long-term antibacterial effect. They must be biocompatible and remove the smear layer without adverse effects on dentin or the sealing ability. Furthermore, they should be low-cost, convenient to use, and not cause any tooth discoloration [3]. They should preferably have the ability to dissolve organic components and inactivate bacterial endotoxins [4]. However, the high properties of irrigating solutions are not enough to achieve desirable disinfection of the canal. Appropriate delivery systems and activation techniques are crucial to fulfilling the objective of chemical debridement. Many techniques and devices were introduced to the dental market but not all of them were effective. Therefore, this comprehensive review aims to summarize the effect of various irrigant activation and agitation techniques or devices on endodontic treatment outcomes. A flowchart of the different irrigant activation and agitation techniques or devices included in this review is shown in Figure 1.

## Review

Manual agitation techniques

Conventional Syringe Irrigation

Syringe irrigation is considered an important technique for its efficiency and wide availability. Therefore, it is the current clinical standard. It is also known as a positive pressure irrigation technique because it is using the force formed inside the barrel due to the pressure on the plunger [5-6]. Various needle types are available with different diameter sizes, tip openings, and flexibility. There are needles made of stainless steel, nickel-titanium [7], and plastic, which enhance flexibility, especially in curved canals. Moreover, there are two available categories of tip opening designs. The first one is open-ended needles that facilitate the direct flow of the irrigant through the tip. The second one is closed-ended needles that facilitate the flow of the irrigant through one or more sides [8]. In open-ended needles, the irrigant will not be able to reach more than 1 mm beyond the tip of the needle when the apical size is prepared to size 30 and should be placed freely 2-3 mm shorter than the working length to avoid irrigant extrusion. Nonetheless, closed-ended needles are considered less effective than open-ended needles in terms of irrigant extension and have less risk of sodium hypochlorite accidents [8-9]. Grossman recognized that adequate apical preparation is needed to enhance irrigation efficacy by conventional syringes [10]. Therefore, clinicians need to balance between effective mechanical irrigation and the size of canal preparation. Moreover, the importance of the needle diameter was noticed to reach the whole root canal system. It is recommended to use small diameter needles ranging from 27-gauge to 31-gauge [4,11]. Mainly 30-gauge needles that correspond to instrument size 35 are considered the standard. The main problem occurs with small diameter needles (less than 30 gauge) that extra force applied on the plunger is needed to ensure the flow of the irrigant [12]. In summary, factors that improve the efficacy of conventional syringe irrigation include proximity of the needle to the apex [13], a large volume of irrigant [14], a small gauge of irrigation needle (30 gauge or less) [13], slow irrigant delivery, and agitation [15]. Nevertheless, inaccessible areas that harbor bacteria and debris were found after conventional syringe irrigation [16]. This issue is happening because of the needle tip location and penetration depth of the irrigating solution. In conclusion, a better system that provides deeper penetration with less apical extrusion and more thorough canal debridement is needed.

Manual Dynamic Agitation (MDA)

Manual dynamic agitation, which is achieved by in and out vertical movements of irrigation needle, fitted master gutta-percha cone, or stirring movement by endodontic files may facilitate irrigant penetration to the full working length by producing a hydrodynamic effect. This has to be reached using 100 strokes of push-pull motion of the gutta-percha point per 30 seconds [17]. Stojicic et al. found that irrigant agitation using active needle irrigation and sonic and ultrasonic activation had the same effect in increasing tissue dissolution speed by sodium hypochlorite up to 10 times when compared with passive irrigation. This concludes that irrigant agitation and refreshment are the main factors to achieve effective cleaning [18].

Endodontic Microbrushes

Microbrushes were introduced to agitate the irrigant and debride canal walls. Some of them also included being indirectly involved in irrigant delivery, such as NaviTip FX (Ultradent Products Inc, South Jordan, UT), which is a 30-gauge irrigation needle tip covered with a brush. However, using these brushes showed an insignificant cleaning effect in the apical and middle third. This result might be improved if an in-and-out scrubbing motion was used during irrigation, but this might lead to bristle dislodgment inside the canal [19].

Machine-assisted irrigation

Rotary Microbrushes

Active brushing was introduced in the 1990s, known as Endobrush (C&S Micro instruments Ltd, Markham, Ontario, Canada). Later, CanalBrush (Coltene Whaledent, Langenau, Germany) was introduced as a highly flexible endodontic microbrush that can be used manually or with a rotary action. However, it is more efficacious when used at a speed of 600 revolutions per minute (RPM). Weise et al. showed that the use of the small and flexible CanalBrush with an irrigant removed debris effectively from simulated canal extensions and irregularities [20] but Protogerou et al. recommended using it with greater canal preparations or smaller size brush for a longer time [21]. A study claimed that this type of brush reaches inaccessible areas in the root canal and has a better removal of tissue and debris when compared to instrumentation alone. However, it showed that the relative size of the brush limits its reach to full working length and can cause packing of debris, especially in the apical part of the root canal [22]. Ruddle improved the idea of these brushes by producing a new series of endo brushes that have appropriate sizes and tapers. Moreover, the diameter, length, and stiffness of the bristles were optimized. Two types were designed: one attached to the rotational 300 RPM flexible plastic core and the other one activated with ultrasonic devices [23]. Unfortunately, Ruddle brushes are not available in the market until the present.

Pressure alternation devices

Apical Negative Pressure Irrigation (ANP)

The EndoVac system: The apical negative pressure (ANP) delivery technique consists of a master delivery tip that delivers the irrigant to the pulp chamber and vacuums the excess, a macro-cannula that suctions irrigants up to the middle segment of the canal, and a micro-cannula that is placed 0.2 mm from the apex to enhance the delivery of the irrigant to the apical part by forming a negative pressure through the multiples micropores to ensure thorough cleaning [24]. Studies found that this technique will decrease the risk of a sodium hypochlorite accident [25], and remove the air entrapped in the apical third of the root canal, which is also known as an apical vapor lock. The apical vapor lock phenomenon that occurs when air is entrapped due to the introduction of liquid in a closed-end microchannel like the root canal, will preclude the apical portion of the canal from directly contacting the irrigant solution, but within hours and days, the canal will be flooded with irrigant. However, this is not applicable during the limited time frame of endodontic treatment. So, techniques to overcome this effect were introduced [26].

The apical negative pressure delivery technique has some limitations when applied clinically such as the requirement of large apical preparation till size 40 to introduce the cannula, which might not be applicable in curved canals [5], cannula blockage with debris [27], and considerations that have to be taken in coronal preparation to introduce the system correctly. A study concluded that within the same time frame, the irrigation volume used with the EndoVac system is much higher than the irrigation volume delivered with syringe irrigation [24]. There are claims that apical negative pressure irrigation had a better cleaning effect than conventional syringe irrigation [24,28] but not enough evidence to support it [29]. In a recent systematic review, apical negative pressure showed a superior effect to conventional syringe irrigation in reducing bacteria, inflammatory infiltrate, and improving periapical healing, but heterogenicity seen in the articles suggests that these results and superiority of a particular irrigation technique are inconclusive [30]. However, studies support that ANP had an advantage in decreasing the risk of irrigant extrusion beyond the apical foramen when compared with syringe irrigation [31].

The Rinsendo System

The Rinsendo system (Dürr Dental Se, Höpfigheimer, Bietigheim-Bissingen, Germany) is another device based on pressure suction technology. It works by hydrodynamic activation of the irrigant and an ultra-thin flexible cannula that is introduced to the apical third of the root canal to ensure negative pressure irrigation. It provides an exact flow rate by dispensing 6.2 ml of irrigant solution per minute using a maximum of 5 psi air pressure to deliver it [32]. This feature enhances irrigant penetration. There are 100 times pressure/suction cycles per minute. The pulsating nature of fluid flow observed an increase in the risk of apical extrusion [33]. An in vitro study found that Nintendo showed less effective stained collagen removal from the root canals in comparison to manual dynamic agitation using a fitted gutta-percha cone. However, no study showed its superiority in chemical debridement or its effect on treatment outcomes [34].

Ultrasonic activation

This is the activation of the irrigant solution inside the root canal using ultrasonic energy between 25-32 Kilohertz (kHz) [35]. This ultrasonic activation technique is considered a widely used technique and a clinical standard [36]. This technique enhances chemical debridement by forming acoustic microstreaming, which is the circular, rapid movement of the irrigant around the vibrating file, and acoustic cavitation, which is the creation and distortion of bubbles. This high energy ensures irrigant flow to remote areas involved in the complex root canal anatomy [37]. The best outcome of ultrasonic activation can be reached when the file acts freely inside the root canals. Moreover, the thinner files showed higher frequency, better streaming velocity, and acoustic microstreaming. Some studies said that the benefit of acoustic cavitation is minimal in ultrasonic irrigation and maybe it doesn’t occur at all [38]. Two types of ultrasonics were described and investigated by researchers. The first one is ultrasonic instrumentation (UI), which is known as a combined irrigation and instrumentation technique. Some studies showed that it results in cleaner canals than those prepared with conventional instrumentation [39-40]. However, other studies showed less efficiency in pulp tissue removal than passive ultrasonic irrigation and the cause as uncontrolled cutting of dentin that might produce strip perforation and highly irregular shaped canals [41-43]. Therefore, ultrasonic instrumentation is not recommended to be used anymore [39]. The second one is passive ultrasonic irrigation (PUI) that consists of irrigation activation without simultaneous instrumentation. This technique transmits energy from the oscillating file to the irrigant inside the root canal to form acoustic streaming and cavitation of the irrigant [38]. Two flushing methods can be used during PUI, namely, continuous ultrasonic irrigation and intermittent ultrasonic irrigation. In intermittent ultrasonic irrigation, a syringe is used to deliver the irrigant into the root canal. This technique allows control of the irrigant amount used and the penetration depth inside the root canal while with continuous ultrasonic irrigation, this is uncontrolled due to continuous irrigant flow through the ultrasonic device itself. Both techniques showed equal effectiveness in dentin debris removal from the root canal in the ex vivo model when the irrigation time was three minutes [44].

Continuous Passive Ultrasonic Irrigation

Continuous irrigant flow during ultrasonic irrigation is highly desirable to improve chemical debridement. One of the devices that uses this concept is Nusstein’s needle-holding device, which enables the attachment of a 25 gauge needle instead of an endosonic file to the ultrasonic handpiece and ensures powerful irrigant activation without breakage of the needle. It also delivers the irrigating solution to the canal continuously through a tube [45]. In vivo studies showed a significantly cleaner canal and isthmuses for both vital and necrotic teeth when used for one minute [46-47]. It also decreased the colony-forming units counted in necrotic teeth [48]. These results might be due to continuous fresh irrigant flow. Moreover, this technique decreases ultrasonic irrigation time [43,49]. Ultrasonic needles are another form of continuous ultrasonic irrigation. It is a 25G open-ended stainless-steel needle. It is known commercially as ProUltra PiezoFlow by Dentsply Sirona, Charlotte, NC. It can be attached to an ultrasonic handpiece and syringe at the same time. The manufacturer recommends using it only in teeth with a closed apex [50]. Studies showed the superiority of this device regarding the removal of pulp remnants and hard tissue debris when compared with conventional syringe irrigation [46,51-52].

Intermittent Passive Ultrasonic Irrigation

Various companies provide intermittent ultrasonic irrigation with different brand names. The main idea behind this technique is the file design of an ultrasonic irrigation device. Stainless steel and nickel-titanium files are used with different tip types, sizes, and tapers. Smooth non-cutting wires are preferable to avoid dentin removal but it showed that some dentin removal happens even when smooth wires are used [53]. Three types of files are generally used in this technique, including smooth wire (Endo Soft Instrument (ESI), electro Medical Systems, Nyon, Switzerland), k-file (Acteon Satelec, Merignac, France), and the Irrisafe file (Acteon Satelec, Merignac, France) [53]. Some of the wires are tapered like ultrasonic K files with a square cross-section that forms sharp cutting edges along with the file. The taper of K files showed lower oscillation amplitude from its free end to the handpiece. Other wires were introduced by Acteon Satelec, Merignacv, and the so-called “Irrisafe”. It has a non-cutting thread with a large pitch, no taper, and a blunt working end with two different lengths and tip sizes [35].

Many studies indicated that passive ultrasonic activation did not remove the smear layer [54]. However, the study by Cameron et al. studies concluded that using 3% sodium hypochlorite (NaOCl) with PUI removes the smear layer [55-56]. Other studies showed a failure to remove the smear layer in the apical third of the canal when ethylenediaminetetraacetic (EDTA) with PUI or a combination of EDTA and NaOCl with PUI were used [57-59]. Two main factors attributed to the success of PUI. The first one is the high ultrasonic power that causes de-agglomeration of bacterial biofilms by acoustic microstreaming. The second one is cavitation that produces bacterial cell wall weakening. These two factors increase the susceptibility of bacteria to the antibacterial effect of sodium hypochlorite [60].

In conclusion, ultrasonic irrigant activation didn’t show superiority to conventional syringe irrigation in the main root canal [12]. However, it showed a better cleaning to challenging areas such as fins, isthmuses, oval canals, accessory or lateral canals, and the apical portion of the curved canals [61-62]. Moreover, passive ultrasonic irrigation is more effective in removing pulp tissue remnants, dentine debris, and planktonic bacteria. It also enhanced the NaOCl-dissolving capacity to organic material by agitation and temperature [63]. Limited studies showed that ultrasonic irrigation has a better antibacterial effect and improved healing when compared with syringe irrigation. So, it remains unclear if ultrasonics irrigation can decrease more microbial load in vitro than syringe irrigation [64]. However, a study concluded that irrigation with a syringe during instrumentation, followed by 10% EDTA and finalized with passive ultrasound irrigation is an effective way for cleaning root canals, independently of the use of chlorhexidine or NaOCl as the final irrigant [65].

Sonic agitation

Sonic agitation uses low sonic energy that ranges between 1000 to 6000 hertz (Hz) onto files within the canal to generate streaming of the irrigant. It depends on the transverse oscillation on the file tip to agitate the irrigant solution inside the root canal. Different companies released tips and devices with multiple sizes and tapers that utilize this mechanism. Endoactivator (Dentsply Sirona, Charlotte, NC), which has a smooth and highly flexible polymer tip, uses very low frequency (160-190 Hz) to reach oscillation [66]. It has a size/taper of small 15/0.02, medium 25/0.04, and large 35/0.04 [67].

The Vibringe sonic agitation device uses the traditional type of syringe delivery with added sonic vibration [68]. While Respisonic is a sonic device that allows file attachment. Moreover, a recent technology known as EDDY tip (VDW, Munich, Germany) was introduced. It is a polyamide tip powered at a high frequency (up to 6,000 Hz ) by an air scaler. Its tip size is 20 with a 0.05 taper [69].

Many studies failed to find any advantage of Endoactivator in cleaning the main root canals, isthmuses, and fins over syringe irrigation [27,70-72]. However, Endoactivator was less effective when used for the same duration as ultrasonic activation [61,66,73]. On the other hand, the EDDY tip showed an effective cleaning as ultrasonic activation [74]. While other studies showed that the EDDY tip has a similar result to syringe irrigation in terms of antibacterial efficacy [75] and debris removal from isthmuses [76]. In the comparison of ultrasonic activation and sonic agitation, a study showed a superior effect of ultrasonic irrigation in removing residual pulp tissues. However, other studies showed an insignificant difference between various ultrasonic and sonic techniques in organic tissue dissolution from simulated grooves in the root canal when sodium hypochlorite and EDTA were used [74].

In conclusion, irrigation activation using various devices results in better outcomes in one or more parameters when compared to passive irrigation. However, the long-term benefit was negligible in the mean of periapical healing that was achieved by radiographic evaluation [77-78] and the postoperative pain that occurs after two days [79-80]. Studies showed less postoperative pain when Endovac [81], ultrasonics [82], Endoactivator [83], and MDA [84] were used.

Continuous irrigation during instrumentation

*Quantec-E Irrigation Pump* 

The Quantec-E irrigation pump ensures continuous irrigant flow during rotary instrumentation by getting attached to the endodontic handpiece. The purpose of this device was to reach cleaner and smear layer-free canals. However, it failed to remove the smear layer from the middle and apical thirds of the canal but had a statistically significant difference in the coronal third that showed cleaner canal walls, less debris, and more complete removal of the smear layer [85].

Self-Adjusting File (SAF)

The self-adjusting file (ReDent Nova, Ra’anana, Israel) is a file designed as a hollow tube. Its walls are made from a thin nickel-titanium lattice with a rough outer surface. Thus, it ensures minimal dentin removal in a vertical vibration motion like scrubbing. The irrigation solution is delivered within the filing system throughout the cleaning and shaping process. This is achieved by using the file with two different systems which are the VATEA irrigation pump and the all-in-one Endostation machine [86]. SAF system doesn’t allow control of the apical enlargement, thus limiting the ability of the irrigants to achieve effective and predictable disinfection [87].

Rotary file agitation 

Max Wire Files

FKG Dentaire, La Chaux de Fonds, Switzerland, was the first that introduced the XP-Endo Finisher. It is made of highly flexible Max wires and can work in two phases (Austentinte and Martensite). It has a zero taper with a tip size equal to 25. It has a larger expansion capacity than the XP shaper and can reach upwards to 6 mm in diameter. Moreover, it is reaching untouched regions inside the root canal without removing dentin or changing the canal shape. A recent study concluded that XP-Endo Finisher with an appropriate irrigation protocol failed to make the apical area of the root canals debris-free [88].

Laser activation 

A group of techniques is categorized under laser activation. These techniques rely on optic cavitation by creating and collapsing vapor bubbles that travel to farther areas inside the root canal and heating the irrigant by light [89-90]. Conventional laser endodontic (CLE) utilizes Nd: YAG, Nd: YAP (760-1400 nm), Er: YAG, and Er: Cr (2940 or 2790 nm). The last two systems are using pulse mood and are highly absorbed in water. Laser-activated irrigation (LAI) also uses Er: Cr and Er: YAG to deliver laser energy inside the root canal by an end or radial firing tip placed deep inside the apical third of the canal [91]. More common laser irrigation used nowadays is photon-induced photoacoustic streaming (PIPS) that has a modified tip placed submerged in the irrigant at the chamber of the root canal and uses short low-energy pulses (50 μs, 20 mJ) at a rate of 15 Hz [92] and repeated three to four times for 20 to 30 seconds each time [93]. Shockwave-enhanced emission photoacoustic streaming (SWEEPS) is the most recent addition to laser irrigation. It has similar features as PIPS and differs in that laser pulses are delivered in pairs forming a primary and secondary cavitation effect with a period of time of approximately 600 μs in between each pulse. It is believed that SWEEP will amplify the secondary cavitation to reach farther areas of the root canal. Another classification under laser irrigation is photoactivated disinfection (PAD), which is achieved with a diode laser that is absorbed by photoactive colored substances (toluidine blue). This technique produces reactive oxygen species and singlet oxygen leading to microbial cell damage. It decontaminates up to 500-750 μm of distance from the main canal. Moreover, it can be used as low-level laser therapy that bio-modulates many cellular functions as anti-inflammatory and analgesia when the correct dose is applied [94].

LAI showed better activation when used for 20 seconds in comparison to ultrasonic activation with regards to the removal of hard tissue debris and biofilm [89,91]. The effect of both techniques can be the same if the duration of activation increases [95]. On the other hand, Miera et al. found that NaOCl was the most effective in *Enterococcus faecalis* removal when compared with Er: YAG, which also resulted in a great decrease in viable counts. However, antimicrobial photodynamic therapy showed weak bacterial reduction [96]. Furthermore, PIPS decreased the time to one minute to get 100% disinfection while 83% of disinfection can be achieved in 20 minutes when conventional needle irrigation is used. So, the most efficient way to use PIPS is to combine it with 6% NaOCl to inhibit *Enterococcus faecalis* [97-98]. However, there is not enough evidence that PIPS has a better effect than LAI [95,99], and it may have a similar antimicrobial effect to syringe irrigation when sodium hypochlorite is used [100]. Another study showed that it has a superior effect in biofilm removal when compared with PUI [101]. A study found that LAI is more effective than PIPS in biofilm removal [91]. Moreover, LAI with NaOCl and EDTA treatment was the most effective in removing the smear layer from the entire root canal wall [102]. Moreover, a randomized clinical trial investigated the difference between laser and ultrasonic activation effects in periapical healing of teeth with chronic apical periodontitis and found that both increase the predictability of endodontic treatment success [103] while another study suggested otherwise [104].

Finally, irradiation protocols used in laser activation have to be interpreted with special care regarding the thermal increase inside the root canal system and the surrounding tissues [105]. A recent systematic review evaluated various laser disinfection in root canal treatment and concluded that there was a lack of standardized protocol and discrepancy in methodologies use. So, they suggested that further investigation has to be done to achieve optimal outcomes [106]. However, Er: YAG, LAI, and PIPS are promising in canal disinfection, debris, and smear layer removal [107-108].

Multisonic activation

Multisonic activation is considered one of the most recent techniques used in chemical debridement. It uses the implosion of bubbles created with acoustic waves in various frequencies to improve irrigant flow all over the root canal system and enhances the chemical effect of the irrigant [109]. GentleWave (Sonendo, Laguna Hills, CA) is the device that uses this technology. It delivers a stream of treatment solution, including EDTA, NaOCl, and distilled water, from the handpiece tip into the pulp chamber while excess fluid is simultaneously removed by the built-in vented suction through the handpiece. First, fluid is optimized by removing undesirable gases. Then, it travels to reach the soundbar housed inside the handpiece. In this part, acoustic energy is released and useful cavitation forms microscopic bubbles that travel to microscopic spaces of complex anatomies and dentinal tubules. This technique ensures minimal shaping of the canals. So, enlarging to an apical size of 15-25 is more than enough. The whole cleaning period takes between five and eight minutes [110].

Multisonic activation showed better results in achieving cleaner isthmuses than syringe irrigation and a superior microbial load reduction in comparison to ultrasonic activation [111]. Moreover, a study concluded 97% successful healing in the vital teeth and teeth with apical periodontitis treated with the GentleWave system at 12 months [112]. However, no control was found in this study. A recent study showed that GentleWave is not significantly better than ultrasonic activation in regards to debris removal [113].

## Conclusions

This comprehensive review concluded that using mechanical active irrigation, which enables the activation or agitation of the irrigating solution, is beneficial in root canal treatment by increasing the efficiency of delivering the irrigant up to the working length and ensuring isthmus cleanliness. However, considerable variation was noticed between the protocols used in each technique, with a lack of designed randomized clinical trials to focus on the long-term outcome of different techniques and their effect on increasing the success of root canal treatment or periapical healing. Moreover, a low level of evidence was noticed regarding the effectiveness of certain activation techniques over others. Therefore, each study needs to be carefully weighed before using its result and embracing its conclusion. Future studies need to focus more on the antimicrobial effect of each technique and its effect on the healing of apical periodontitis. Also, recent advances, such as multisonic and laser activation, are promising tools that need more clinical investigations to show their efficiency.
